# Interventions to develop collectivistic leadership in healthcare settings: a systematic review

**DOI:** 10.1186/s12913-019-3883-x

**Published:** 2019-01-25

**Authors:** Aoife De Brún, Roisin O’Donovan, Eilish McAuliffe

**Affiliations:** School of Nursing, Midwifery & Health Systems, Health Sciences Centre, University College Dublin, Dublin 4, Ireland

**Keywords:** Collectivistic leadership, Collective leadership, Shared leadership, Healthcare, Systematic review

## Abstract

**Background:**

Collective or shared leadership approaches have been associated with team performance outcomes in several sectors. Based on this evidence, there have been calls for more inclusive approaches to leadership in healthcare settings, but guidance on how to achieve collective leadership is lacking. This study synthesised knowledge of interventions to introduce collectivistic leadership in healthcare settings.

**Methods:**

The databases of PubMed, PsychInfo, ABI Inform, Cochrane and CINAHL and three grey literature databases were searched. Studies from any country were included if they reported on the development and evaluation and/or implementation of training/interventions to develop collectivistic leadership and reported individual and/or team-level outcomes. Results were synthesised using a narrative approach.

**Results:**

The searches yielded 4448 records of which 21 met the eligibility criteria and were reviewed. Studies used a variety of interventions; eleven employed a team training approach, four described co-leadership, three explored service improvement, two detailed co-design approaches and one described an individual team development intervention. Most demonstrated moderate to good success in enabling collectivistic leadership, with benefits reported in staff engagement, satisfaction, and team performance.

**Conclusions:**

Whilst collectivistic leadership interventions have demonstrated positive outcomes, there is a need for more rigor and consistency in the evaluation of interventions aimed at developing collectivistic leadership approaches in health settings.

**Electronic supplementary material:**

The online version of this article (10.1186/s12913-019-3883-x) contains supplementary material, which is available to authorized users.

## Background

Traditionally, the concept of a leader and of leadership has focused on individuals, where leaders are trained to lead a work group or team as part of a hierarchical organisational structure. Accordingly, leadership research has conventionally focused on vertical leadership and leader-follower interactions, where one focal leader interacts with subordinates or followers in dyads or small groups with clear lines of authority and power across levels of the hierarchy [1, 2]. Leadership has been considered as an individual attribute, and contested as either trait-like or a state-like quality, or both [3, 4]. This body of research has focused on the single leader, with the team or group outputs (e.g., performance, quality targets) and staff outcomes (e.g., satisfaction, engagement) considered as reflective of leader effectiveness. Gibb, in 1954, was among the first to recognise the potential for leadership at the group or team level, rather than at the individual level, asserting that “leadership is probably best conceived as a group quality, as a set of functions which must be carried out by the group” ([5]; 884). However, it is only more recently that collectivistic or distributed approaches to leadership have garnered increased attention [1, 6].

Collective approaches to leadership are evident where the leadership roles and responsibilities are shared, distributed or rotated amongst team members. Various forms of collectivistic leadership exist, including distributed, shared, team, co-leadership, rotated, and collective leadership, to name a few. Distributed leadership [7, 8], for instance, is indicated by various patterns of distributed roles and responsibilities shared among multiple individuals where there is conjoint action by the group. Similarly, shared leadership has been described as “an emergent team property that results from the distribution of leadership influence across multiple team members” [9]. Friedrich et al. characterise collective leadership as “a dynamic leadership process in which a defined leader, or set of leaders, selectively utilise skills and expertise within a network, effectively distributing elements of the leadership role as the situation or problem at hand requires” ([10]: 933). Central to collective leadership is the principle that team members interact to lead the team by sharing in leadership responsibilities at different times [11, 12]. Although the various terms represents distinct theoretical approaches, even with multiple proposed frameworks within each approach [13], it is important to note that often the terms have been used interchangeably, and the relative usage of terms has varied over time [13]. Ultimately however, these forms of leadership defy the traditional, hierarchical, single leader view of leadership and represent a shift towards these more collectivistic approaches.

A meta-analysis of shared leadership and team effectiveness developed a composite definition for these various forms of shared leadership as “an emergent and dynamic team phenomenon whereby leadership roles and influence are distributed among team members” ([14]: 5). For the purposes of this review, we use the term ‘collectivistic leadership’ to include various non-traditional approaches to leadership, including shared, distributed, collective, and related or similar concepts, that share the roles and responsibilities across more than one member of a work group or team over time, through both formal and informal mechanisms [1, 15].

Since the 1990s, there has been increased interest in collectivistic approaches to leadership [14] and there is accumulating evidence of the positive impact of such approaches from many settings. Two meta-analyses exploring the impact of collective, shared and/or distributed leadership in teams have found that, across sectors, shared leadership predicts team effectiveness and team performance outcomes [14, 16]. Furthermore, shared leadership has been found to be a better predictor of team performance and organisational outcomes than vertical leadership structures [17, 18].

In a recent review of the evidence base for leadership in health settings, it was concluded that “leadership is the most influential factor in shaping organisational culture and so ensuring the necessary leadership behaviours, strategies and qualities are developed is fundamental to health services improvement” [19]. Research has linked effective leadership behaviours in a health setting to quality and safety and patient outcomes [20] and has highlighted poor leadership as potential causal factors in patient safety failures [21]. Furthermore, leadership with a strong emphasis on hierarchy potentially inhibits a positive safety climate due to fear of blame and repercussions for reporting safety-related problems [22]. Research in the UK has indicated that the best performing hospitals were those in which staff demonstrated high levels of engagement in decision-making and where there was evidence of distributed leadership in the organisation [23]. Given this emerging evidence base, there have been calls to move from traditional models to shared and distributed models of leadership in healthcare settings [6, 8, 19], where increasingly, care is delivered via multidisciplinary teams.

Whilst collectivistic approaches to leadership have been linked with positive outcomes, there is little guidance on how best to introduce and develop collective leadership in practice. The field is still developing and as a result, the literature is sparse and disparate; thus, we do not yet know how we can effectively develop collectivistic leadership approaches [1]. Furthermore, because such approaches are relatively novel in healthcare settings there is a lack of understanding on how best to achieve collective ways of working in this context. To address this gap and to inform future research in this area, this paper aims to synthesise scientific knowledge of evaluated interventions that sought to introduce collectivistic leadership in healthcare settings. The paper provides insight into the type and content of interventions that have been designed and tested, their theoretical underpinnings, means of evaluation, and efficacy. The aim of this systematic review is to address the following research question: *What interventions are the most effective for the development of collective leadership in healthcare teams, what outcomes have been measured, and what evaluation approaches have been adopted?* We are interested in exploring both the means of evaluation and evaluation outcomes to explore interventions designed to enhance the practice of collectivistic forms of leadership. As discussed above, the terms ‘collectivistic’ will be employed in this review as a broad term to capture the various forms of non-hierarchical approaches to leadership.

## Methods

### Systematic reviewing

A systematic review was conducted to explore the topic. Systematic reviewing is a method to synthesise the available scientific evidence to address a clearly formulated research question. It enables researchers to collate relevant studies, assess the quality of evidence, and generate conclusions and/or identify knowledge gaps. The current review employed methods informed by Cochrane guidance on conducted reviews [24] and results are reported in line with the Preferred Reporting Items for Systematic Reviews and Meta-Analyses (PRISMA) guidelines [25, 26]. The protocol for this systematic review was published in the PROSPERO Database in May 2017 (reference: CRD42017065007).

### Search and section strategy

As recommended by the Evidence for Policy and Practice Information and Co-ordinating Centre [27], the search strategy attempted to balance sensitivity with specificity in its results. Initial scoping searches revealed that the search strategy was highly sensitive and returned large numbers of studies not relevant to the topic. This indicated that MeSH terms were too broad for the purposes of this review. Instead keywords were used to ensure more specificity in the search. Previous systematic reviews conducted on collectivistic approaches to leadership from other sectors helped to inform the search strategy [14, 16]. The electronic databases of PubMed, PsychInfo, ABI Inform, Cochrane and CINAHL were searched on 8-10th February 2017 to find relevant studies. The complete search strings are included in Additional file 1. Some of the keywords and terms used included ‘collective leadership’, ‘shared leadership’, ‘distributed leadership’, ‘health’, ‘clinical’, ‘intervention’, and ‘training’. Groups of keywords relevant to a specific category (for instance, setting) type were combined using the ‘OR’ Boolean term (e.g., health OR clinical OR medical) and categories of keywords were then combined using the AND Boolean operand (see Tables 1 and 2). The search strategy was reviewed by a researcher with extensive systematic reviewing experience who was not involved in the study.

**Table 1:** Database search results

Database	Population	Intervention focus Intervention		Setting	Combined search results
	Team* Group*	Collective leadership Collectivistic leadership Distributed leadership Shared leadership Collaborative leadership Participatory leadership Inclusive leadership Democratic leadership Plural leadership Dispersed leadership Empowering leadership Compassionate leadership Informal leadership Peer leadership Team leadership	Skill* Intervention* Development Education* Training Strateg* Program* Module* Course* learning framework* competenc* capabilit* model* curricul* e-learning workshop*	Health healthcare medical clinical nursing hospital primary care community	
PsychInfo	509,397	10,447	1,363,982	782,683	1904
PubMed	2,125,773	532	4,477,818	3,549,729	216
ABI Inform	887,718	17,550	2,298,042	674,818	1304
Cochrane	6976	130	9186	9232	118
CINAHL	402,321	1227	960,749	1,185,768	539
TOTAL					4081

* Represents the use of the 'wildcard' Boolean truncation symbol to return all results with this root, and can represent any number of letters following this in the word, e.g., group. Will retrieve records relating to group, groups, groupings, etc

**Table 2:** Grey literature search results

Grey literature search engine	Search strategy	No. of items screened	No. of items for full text review	No. of items included
OpenGrey	collective leadership OR shared leadership OR distributed leadership AND healthcare	77	0	0
OpenDOAR	collective leadership OR shared leadership OR distributed leadership AND healthcare	First 100 records (results sorted by relevance)	3	2
OAIster	kw: collective leadership OR shared leadership OR distributed leadership kw: healthcare	160	2	0
Other (hand searching and studies identified through experts)	[various]	30	5	2
Total		367	10	4

No restrictions were placed on country of origin or language, although searches were restricted to papers, technical reports, and accessible dissertations published since 2000. The academic database searches were supplemented with searchers of grey literature databases OpenDOAR, OpenGrey and OAIster. These databases are amongst those recommended due to their broad scope and their ability to enable specific searches that return the most relevant records [28, 29]. Additionally, the research team scanned the reference lists of included papers and contacted experts in the field to help identify other potentially relevant studies.

### Inclusion/exclusion criteria

Experimental and observational research studies from a healthcare setting, including quantitative, mixed methods and qualitative studies related to plural or collectivistic forms of leadership (shared, collective, distributed leadership, etc.) from any country were eligible for inclusion in the review. Studies were eligible if they reported on the development, evaluation and/or implementation of training or interventions to foster collectivistic approaches to leadership. Studies had to report individual and/or team level changes related to leadership roles and responsibilities, experiences of working, or health, team, patient, safety outcomes or outcomes relevant to team performance or team effectiveness. Given the potential range of variables and outcomes across different healthcare settings, specific outcome measures were not predetermined.

### Study screening and data extraction

The online specialised systematic review website, Covidence, was employed to manage the review [30]. Covidence enables two reviewers to independently screen records, it displays conflicts and tracks the number of papers excluded and reason for exclusion at each phase of the systematic review. Two reviewers independently screened record titles and abstracts based on the eligibility criteria. Where there was any disagreement or ambiguity, a third reviewer assessed the relevant records and consensus was reached on eligibility through discussion, and, where appropriate, retrieval and review of the full-text document.

A structured data extraction form to capture information from the relevant records was developed. Consistent with recommendations for best practice for systematic reviews of interventions [31, 32], the data extraction template collected information relevant to: study details (country of study, setting/context, sample size); theory, framework or model underpinning the intervention; mode of delivery of intervention; the specific content of intervention(s); duration of intervention(s); measures/variables of interest used to evaluate impact of interventions; outcomes of interventions/training programmes related to: impact on leadership (roles and responsibilities) for individuals and teams, impact on individual/team performance, other measured outcomes (likely to vary between studies and therefore not stated in advance); and reported/hypothesised determinants of (non-)effectiveness of interventions/programmes. Where information was missing or incomplete in studies included in the review, efforts were made to contact authors and/or funders to access further details. The data extraction form was hosted on SurveyMonkey and allowed both reviewers to extract data independently and enabled comparisons between reviewers.

### Quality appraisal

As appropriate to the study design, methodological quality assessment frameworks including an adapted version of the CASP Quantitative cohort study tool [33], the Critical Appraisal Skills Programme (CASP) Qualitative tool [34], and the Mixed Method Appraisal Tool (MMAT) [35] were used to evaluate the quality of included studies. These tools have been widely used and are considered a valid indicator of methodological quality. Due to the small number of relevant studies that met the inclusion criteria and the variety of study designs included, no studies were excluded from the review based on the quality assessment outcome.

### Data synthesis

As this review includes studies with large heterogeneity in interventions and measures, a statistical analysis was inappropriate. Results were synthesised using a narrative approach [36] and results reported in accordance with PRISMA guidelines [25, 26] and current recommendations on the description of interventions in systematic reviews [31].

## Results

### Search results

**Fig. 1:**
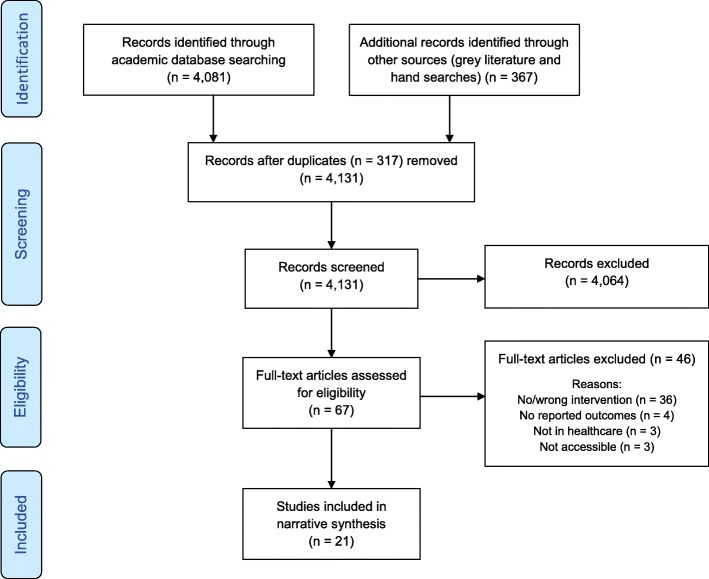
PRISMA flow diagram

The databases searches yielded a total of 4448 studies. Of these, 317 duplicates were removed, 4064 were excluded after title and abstract screening and a further 46 were excluded for reasons including not providing a sufficiently detailed description of the intervention, not taking place in a healthcare setting and/or not reporting an intervention outcome. Figure 1 depicts the PRISMA flow chart and summarises the screening and selection phases of the review process. In total, 21 studies met the eligibility criteria and were included in the review.

### Overview of included studies

Table 3 summarises the studies included in the review and details the study characteristics, including year published, study location, aim, participant details, methodological characteristics and key findings. Of the 21 studies, four were found through grey literature search engines and these four represent work conducted as part of a doctoral dissertation. The majority of included studies were conducted in the USA [37–46] and the UK [47–50] with studies from Australia [51, 52], Sweden [53, 54], Canada [55, 56], and Germany [57] also included. Primarily, these studies were conducted in hospital settings [37, 39–47, 50, 53–57], with one taking place in primary care [38] and across other health settings [51, 52] or multiple sectors [48, 49]. Eleven of the studies used a team training approach [38, 41–44, 48–52, 55], four described co-leadership interventions [46, 54, 57], three explored service improvement interventions [37, 45, 47], two detailed co-design interventions [39, 40], and one related to an individual team development intervention [56].

**Table 3:** Summary characteristics of included studies

Author, Year	Aim	Participants (setting, location)	Underpinning Theory	Intervention Duration	Intervention Content	Methods of Evaluation (Evaluation approach)	Key Findings
MacPhail et al., 2015 [51]	To increase staff willingness to take on leadership roles	11 in first round, 20 in second round (regional healthcare centre, Australia)	Transformational, distributed and systems-based leadership	9–10 months; 2-h sessions on-site once a month (~ 20 h total)	Sessions included a guest speaker and group discussion; one self-organised external site visit and one mini-project in small, interdisciplinary groups; and a presentation to peers and executive staff of learning.	Survey on staff willingness to take on a leadership role; new leadership roles after 18 months; Senior executive feedback on programme (Mixed methods)	‘The clinical leadership programme significantly increased willingness to take on leadership roles … (93%) reported that they were more willing to take on a leadership role within their team.’
Buckley et al. 2009 [37]	To build physician-nurse leadership partnerships based on shared responsibility and accountability for increasing quality and patient safety	28 people across 7 clinical care teams (hospital setting, USA)	None reported	One-month pilot period	Consultants partnered with teams to help build skills in goal-setting, managing staff, promoting positive changes in work culture, and negotiating and resolving conflicts. Team members learned how to structure projects, collect and analyse data and develop action plans for improvement. To the extent possible, physician and nurse leaders completed training together.	Unclear regarding qualitative findings – no methods reported, just results. Individual improvement project case studies and outcomes provided as examples of efficacy of intervention. (Mixed methods)	‘Resulted in breakthrough improvements in quality and patient safety, but also forged better physician-nurse collaboration and job satisfaction.’ ‘Physicians and nurses also came to better appreciate each other’s pressures and challenges.’
Boak et al., 2015 [47]	To analyse the introduction of distributed leadership and team working in a physiotherapy department, and to explore the factors that enabled success.	26 staff members physiotherapy department (hospital, UK)	Distributed leadership and shared leadership	8 months to plan service re-design	Service re-design with quality improvement. Restructuring to speciality team-based service delivery. Co-design of new structures and processes, standardised assessment and treatment protocols, with team member rotation.	Waiting times for routine new patient appointments; Patient Satisfaction; staff experiences of changes (Mixed methods)	Concluded distributed leadership was successfully introduced. Distributed leadership and team working were central to a number of systems changes that were initiated by the department and led to improvements in patient waiting times for therapy. Six factors identified that influenced success.
Gibb et al., 2016 [52]	To standardise and improve team communication and team leadership in care delivered in a residential aged care facility.	4 care units/teams (residential aged care facility, Australia)	Distributed leadership	Not reported	(i) Huddle: 3–5 min ‘time out’ in the work site for shared problem-solving; 4-step protocol. (ii) Giving positive and constructive feedback, using feedback. (iii) Briefing: a leader-facilitated discussion prior to starting shift. Debrief: 3–5 min after-action review (iv) ISBAR to enhance clear communication.	A cultural scan conducted pre-intervention involved the collection of interview, focus group and observational data, with the aim of triangulating data on the current culture, then repeating post intervention to profile changes emerging from the intervention. Short survey pre/post training to measure knowledge, confidence and use of skills. Interviews and focus groups with staff. (Mixed methods)	‘Of the four teams involved in training, only one successfully transitioned to working with the new protocols … distributed leadership was critical to the high performance achieved in Team A.’ Results suggested that the project had more general impact on cultural values and interpersonal behavior and less on the assimilation of standard communication and team working.
Howard et al., 2012 [38]	To deliver a facilitated, team-based quality improvement intervention to encourage leadership inclusiveness.	60 quality improvement teams; 8 of these cases selected and highlighted in paper (primary care, USA)	Inclusive leadership and collaboration	3 months	Used the Multi-Method Assessment Process (MAP) and the Reflective Adaptive Process (RAP). The MAP model was a baseline assessment in which the facilitator-researcher spent 5–7 days observing operations and relationships, interviewing practice members, and developing rapport. This was followed by the RAP, which involved up to 12 weekly, facilitated team meetings with representatives from different areas of the practice. External facilitators modelled inclusive leadership, encouraging reflection and open communication, supporting implementation of improvement plan and discussed principles of inclusive leadership.	Data sources included observational field notes, interviews, and audio-recorded quality improvement meetings to explore exemplar and non-exemplar cases (Qualitative)	Analysis extended case illustrations of 3 physician leadership behaviours that exemplified leadership inclusiveness (explicitly soliciting team input; engaging in participatory decision making; and facilitating the inclusion of non–team members) as well as 3 behaviours that are counter to inclusiveness
Rosengren et al., 2010 [53]	To describe the views of staff after introduction of shared leadership between two nurse mangers for all tasks in the unit.	64/81 of ICU team (hospital, Sweden)	Shared leadership	“a 3-year project”	Introduction of co-leadership model: two leaders to share leadership responsibilities.	Individual’s perception of the work situation; quality of leadership; staff views on shared leadership (Mixed methods)	Staff reported positive views of work and the model shared leadership in terms of confidence and in relation to organizational culture, social interactions, work satisfaction, leadership, Shared leadership & work motives.
Sanders et al., 2013 [39]	To create a shared leadership model (Staff Nurse Council with clinical nurse representation from all departments and services across the hospital) to introduce initiatives to enhance the work environment.	Nursing teams throughout organisation; number not reported (hospital, USA)	Shared leadership	Not reported	Devolving leadership and decision-making to develop shared leadership model where employees were tasked with introducing initiatives to positively shape the work environment	Staff satisfaction and engagement; levels of stress and fatigue; patient satisfaction scores; nurse-physician relationships (Quantitative)	‘A highly engaged, well-educated, and committed nursing workforce, nurtured by a strong leadership team, has created a positive work environment characterised by low turnover and high retention’
Miller et al., 2007 [48]	To support the development of shared leadership in the teams through the intervention of specially trained and supported leadership development consultants who worked with clinical teams delivering diabetes care	6 diabetes teams (primary and secondary care, UK)	Shared leadership; distributed leadership; collective leadership; blended leadership	13 days over 18 months	Process had 3 residential learning sessions which brought the teams together at the outset, in the middle and at the end of the scheme for sharing learning and expert input; and was also in part tailored to teams. Each leadership development consultant worked with their team to agree opportunities to bring teams together to address team processes, and each emerged with an individual schedule of how to use the 30 days allocated over the 18 months of the scheme.	Each team identified its own criteria for success and team members recorded this at regular intervals. The evaluation used range of methodologies including interviews with team members at two separate time points; a questionnaire survey of all team members at two time points; a questionnaire survey of comparator teams at the same time points; interviews with the leadership development; interviews with patients; and a review of national medical data. (Mixed methods – though only qualitative results reported)	‘Strong evidence from the teams that they were working better together … and becoming more effective as teams.’ However, teams remained convinced that every team needs a leader, felt that leadership was not shared. Sense that the intervention had enabled each of them to develop as leaders and made leaders more willing to listen to them.
Steinert et al., 2006 [57]	To create a new shared leadership of medico-therapeutic staff and nursing staff on all clinical levels	131/165 staff members (psychiatric hospital, Germany)	Shared leadership and shared governance	Not reported	Introduction of co-leadership model between medico-therapeutic staff and nursing staff sharing leadership roles and responsibilities	Staff satisfaction with shared leadership (concrete personal experiences and general experiences with the hospital); appeal of adopting a leadership position Quantitative (self-designed survey)	‘Staff members were satisfied with the shared leadership model both in their own clinical practice and in general. Non-medical staff members were significantly more in favour of several aspects of shared leadership than physicians.’ Evidence the model may have advantages in the management of psychiatric hospitals.
Casady & Dowd, 2005 [40]	Service re-design to create new co-facilitated groups to develop strategies and co-design interventions to enhance employee engagement, involvement in decision-making and increase staff retention.	One medical imaging department (hospital, USA)	Shared leadership	Service re-design over 5-year period	Service re-design and re-structuring with new oversight committee and strategic thinking group (two co-facilitators) to co-design initiatives to enhance staff engagement, create more effective leadership, enable greater participation by staff in the decision- making process, and ensure competitive salaries. On-going since 2001.	Gallup poll survey results related to employee turnover/retention, employee engagement and patient satisfaction (Quantitative)	‘Dramatic change in turnover rate as a result of engaging staff using shared leadership principles.’ Turnover rate decreased from 40% in 2001 to 4% in 2004 with improvement in employee engagement from 44th percentile in 2002 to 69th percentile in 2004.
Allen, 2010 [41]^a^	To evaluate the effectiveness of a work based shared leadership training program	12 Unit-based nursing teams (*n* = 39) (hospital, USA)	Shared leadership	2–3 months	Work-based action learning program consisted of four sessions, which included assignment of an action learning project, cognitive instruction, coaching, and reflection.	Self-designed survey instrument measured team dynamic knowledge, use of shared leadership behaviours, and engagement. (Quantitative)	Results indicated the training program was successful in developing shared leadership: significant changes in knowledge of shared leadership, shared leadership behaviours and team engagement were observed.
Roberts, 2009 [42] ^a^	To facilitate network development that bridges disciplinary silos and fosters collective leadership capacity	Leaders, managers, supervisors, team leaders and coordinators (hospital, USA)	Collective and collaborative leadership	Approx. 5–6 months per cohort	3-h sessions every second month for 6 months. 2 versions: *LEAD 1*: Use of personality profiles to develop understanding, session on review and debriefing on leadership, effective communication, coaching for success, delegation and empowerment, time and meeting management, then a final review, reflection, action planning session. *LEAD 2*: Information on servant leadership, appreciative inquiry, change management, and review, reflection and action planning ‘Lunchtime inquiry group’ who met monthly over a five-month period.	Qualitative approach using reflection, interviews, focus groups, field notes and observation to explore collective leadership, inclusiveness, empowerment, collaboration across units, leading at all levels. (Qualitative)	Findings suggest that the intervention can strengthen skills of individual leaders and foster collective leadership
Van Zwanenberg, 2009 [49]	Co-design of new collaborative leadership programme to find ways of achieving application of learning around collaborative leadership and sustainability of learning.	7 teams (multisector mental health teams, UK)	Collaborative / team leadership	12 months	2-day introduction (establishing goals, mentor, selecting projects); 12 x monthly learning sets on personal qualities for leading change; 6 x bi-monthly functional leaning sets focused on personal development, leading change, developing collaborative relationships; e-resources (performance, financial, risk, management and leadership theory); 1-day programme review and evaluation.	Interviews with participants, the project group, and key stakeholders, and questionnaire responses and focus group sessions for both participants and learning set facilitators.(Mixed methods)	‘Positive shifts in participants’ competence as collaborative partners, particularly regarding working across traditional boundaries.’
Dewar & Cook, 2014 [50]	Intervention aimed to support staff to work together to develop a culture of inquiry that would enhance delivery of compassionate care	86 nursing staff members(hospital, UK)	Distributed and collaborative leadership	12 months	Reflective spaces within the programme, community of practice, Action Learning, Work based activities.	Staff culture questionnaire, reflections following action learning, descriptions of reported staff developments, case studies, staff interviews to elicit impact of training (Mixed methods)	‘Enhanced self-awareness, better relationships, greater ability to reflect on practice, different conversations in the workplace that were more compassionate and respectful, and an ethos of continuing learning and improvement … supported participants … to be reflective and engaged.’
Awad et al., 2004 [43]	Curriculum implemented with objectives of training residents to have the capacity/ability to create and manage powerful teams through alignment, communication, and integrity	Surgical residents (number not reported) (hospital, USA)	Collaborative leadership	“over the course of a surgical residency”	Focused program was implemented with objectives of training the residents to have the capacity/ability to create and manage powerful teams through alignment, communication, and integrity while working 80 h per week. Specific strategies were: (1) to focus on quality of patient care/ service while receiving a high education-to-service ratio, and (2) to maximize efficiency through time management.	Pre/post survey assessing resident’s view of leadership in the areas of alignment, communication, and integrity. (Quantitative)	There was a significant increase in the scores with regards to alignment, communication, and integrity after completion of the leadership training program indicating the program was successful in its aims
Jackson, 2000 [55]	Service re-design to share leadership across clinical units	69 individuals within 4 work groups(hospital, Canada)	Shared leadership	Introduced across organisation over two-year period	A group of staff members and managers assembled in 1997 to form a shared leadership resource group, with function of providing support to individuals and teams as they increased their decision making. The shared leadership resource group gave the responsibility of implementing the model on each of the clinical units to the team leader managers/program directors.	Focus groups and interviews to explore staff experiences and perceptions or service re-design(Qualitative)	Drivers and barriers affecting implementation are explored. ‘Internalisation of the concepts specific to the shared leadership model … was vital … Processes and interactions which meet the demonstrated need of all staff to feel valued, unique, connected, and a sense of belonging are desirable when promoting the model.’
Pelayo, 2008 [44]^a^	Service re-design to team-based leadership to enhance effective functioning of hospital	Organisation-wide team-based working, 1700 employees (hospital, USA)	Team-based leadership	Phased shift to team-based model over 8-year period	As described by the current CEO, the strategies are reported in phases: (a) immediate reactionary phase (controlling finances, removing waste in system), (b) building phase (hiring the right people, training them, ensuring that the teams were functioning efficiently), and (c) self-governing phase (teams tasked to develop specific goals aligned to organisation’s goals).	A qualitative case study (interviews, document reviews, observations) exploration of the strategies organisational leaders adopted to develop a 10-year team-based leadership structure. (Qualitative)	Four conclusions on the team-based leadership: (i) phenomenon that occurred over time and evolved from various strategies to address financial challenges; (ii) none of the strategies employed were considered a failure; (iii) multidisciplinary teams positively influenced the business aspect of the organisation’s performance while increasing the quantity and quality of services; and (iv) the organisation’s teams paralleled the functions and goals of the management team.
Klinga et al., 2016 [54]	Service re-design of organisation to promote shared treatment and shared leadership	Organisation-wide integrated health and social care organisation (hospital, Sweden)	Co-leadership	Service re-design in operation since 1995	Service re-design where each centre is managed through co-leadership shared by two equal leaders (‘pair-leadership’), where responsibility of unit management shared by two co-leaders.	Interviews with eight managers exercising co-leadership to identify essential preconditions in fulfilling the management assignment, operationalisation and impact on integration of health and social care.(Qualitative)	Identified contextual preconditions were an organisation-wide model supporting co-leadership and co-location of services. Perception of the management role as a collective activity, continuous communication and lack of prestige were essential personal and interpersonal preconditions. In daily practice, office sharing, being able to give and take and support each other contributed to success.
Swensen et al., 2016 [45]	To develop a qualitative descriptive case study of the Mayo Clinic leadership development philosophy, approach and model; to understand the features of team-based leadership development	Organisation-wide (hospital, USA)	Collective leadership; team-based leadership	On-going (introduction date not reported)	Organisation-wide policies and programmes including leadership programme, rotating leadership positions, and a collaborative leadership structure.	Staff engagement; patient satisfaction; staff turnover; quality outcomes(Qualitative case study using quantitative data as evidence of effectiveness)	Organisational and governance systems are designed to develop culturally aligned leaders, build social capital, grow employee engagement, foster collaboration, nurture collegiality and engender trust.
Black & Westwood, 2004 [56]	To evaluate the effectiveness of a group-based team leadership development workshop	7 (of 9) invited participated. Administration team in cancer care (hospital, Canada)	Team-based (non-hierarchical) leadership	35 h over 3-month period	3 workshops (35 facilitated hours) *Workshop 1* – team building/getting to know team, role play, Amundson individual styles survey *Workshop 2* – Role clarification using enactment, discussion on mistakes/deaths in healthcare, *Workshop 3* – Review group goals, Johari window self-awareness exercise, group communication exercise and discussion, enactment and demonstration of critical team meeting, debrief.	Interviews 3-months post-intervention to elicit information related to their participation in the workshops and communication, morale, support and the ability to provide more holistic support. (Qualitative)	All participants reported understanding others/being understood; formation of connections with others; sense of belonging/ acceptance; sense of safety and trust in communication; appreciation of facilitation of the group; experience of group morale (increased and decreased); reciprocity and demonstration of support.
Senn, 2014 [46]^a^	To explore factors that hindered or enhanced the role development of co-leaders; and the nature and dynamics of the co-leaders’ working relationship.	8 co-leaders (hospital, USA)	Co-leadership	Co-leadership operational for more than 5 years prior to data collection.	Service re-design – nurse and physician co-leadership model, sharing responsibilities and leading a hospital unit	Individual interviews with co-leaders about their shared role and responsibilities, and their collaborative work together within a co-leadership structure (Qualitative)	Two themes emerged: ‘Shared Role Space: Moving from I to We’ and ‘Partnered Leadership: Dynamic Interplay of Complementary Competencies’. Factors that enhanced/hindered the role identified.

Note: ^a^indicates study found through grey literature searches

### Quality assessment

Tables 4, 5 and 6 summarise the results of the quality appraisal of articles using various tools, as appropriate to study design [33–35]. Tables are arranged to list the studies in order of quality appraisal, with those of higher quality appearing first.

**Table 4:** Results of CASP Qualitative Checklist

Author, Year	Statement of aims	Qualitative methodology appropriate	Research design appropriate	Recruitment strategy appropriate?	Data collection appropriate	Relationship between researcher and participant considered	Ethical issues considered	Data analysis rigorous	Statement of findings	Is research valuable	Outcome of checklist (Yes/Can’t tell/ No)
Roberts, 2009 [42]	Yes	Yes	Yes	Yes	Yes	Yes	Yes	Yes	Yes	Yes	10/0/0
Senn, 2014 [46]	Yes	Yes	Yes	Yes	Yes	Yes	Yes	Yes	Yes	Yes	10/0/0
Howard et al., 2012 [38]	Yes	Yes	Yes	Yes	Yes	Can’t tell	Yes	Yes	Yes	Yes	9/1/0
Klinga et al., 2016 [54]	Yes	Yes	Yes	Yes	Yes	Can’t tell	Yes	Yes	Yes	Yes	9/1/0
Black & Westwood, 2004 [56]	Yes	Yes	Yes	Yes	Yes	Can’t tell	Can’t tell	Yes	Yes	Yes	8/2/0
Pelayo, 2008 [44]	Yes	Yes	Yes	Yes	Yes	Can’t tell	Yes	Yes	Yes	Can’t tell	8/2/0
Jackson, 2000 [55]	Yes	Yes	Yes	Can’t tell	Yes	Can’t tell	Can’t tell	Yes	Yes	Yes	7/3/0
Swensen et al., 2016 [45]	Yes	Yes	Yes	Can’t tell	Can’t tell	Can’t tell	Can’t tell	Can’t tell	Can’t tell	Can’t tell	3/7/0

**Table 5:** Results of CASP Quantitative Study Checklist

Author, Year	Focused issue addressed	Acceptable recruitment	Exposure measured to minimise bias	Outcome measured to minimise bias	Confounding factors identified	Design/ analysis considers confounding variables	Follow up of subjects complete enough	Follow up of subjects long enough	Results believed	Can results be applied to local population	Results fit with other evidence	Outcome of checklist (Yes/ Can’t tell /No)
Allen, 2010 [41]	Yes	Yes	Yes	Yes	No	No	Yes	Yes	Yes	Yes	Yes	9/0/2
Casady & Dowd, 2005 [40]	Yes	Can’t tell	Yes	Yes	No	No	Yes	Yes	Yes	Yes	Yes	8/1/2
Awad et al., 2004 [43]	Yes	Yes	Yes	Yes	Can’t tell	No	Can’t tell	Yes	Yes^a^	Can’t tell	Yes	7/3/1
Steinert et al., 2006 [57]	Yes	Yes	Can’t tell	Yes	No	No	Yes	Yes	Yes	Can’t tell	Yes	7/2/2
Sanders et al., 2013 [39]	Yes	Can’t tell	Can’t tell	Yes	No	No	Yes	Yes	Can’t tell	Can’t tell	Yes	5/4/2

^a^Limited information given on components of intervention

**Table 6:** Results of Mixed Methods Appraisal Tool (MMAT)

Author, Year	Clear objective	Data relevant to addressing objectives	Qualitative data sources relevant to objectives	Qualitative data analysis relevant to objectives	Consideration given to how findings relate to context	Consideration given to how findings relate to researchers’ influence	Sampling strategy relevant to address quantitative research question	Sample representative of population	Quantitative measure appropriate	Acceptable response rate^a^	Mixed methods design relevant to objective	Integration of qualitative and quantitative relevant to objective	Limitations of integrating findings considered	Outcomes of checklist (Yes/Can’t tell/No)
Dewar & Cook, 2014 [50]	Yes	Yes	Yes	Yes	Yes	Can’t tell	Yes	Yes	Can’t tell	Yes	Yes	Yes	No	10/2/1
Rosengren et al., 2010 [53]	Yes	Yes	Yes	Can’t tell	Yes	Can’t tell	Yes	Yes	Yes	Yes	Yes	Yes	No	10/2/1
MacPhail et al., 2015 [51]	Yes	Yes	Yes	Can’t tell	Can’t tell	Can’t tell	Yes	Yes	Can’t tell	Yes	Yes	Yes	No	8/4/1
Boak et al., 2015 [47]	Yes	Yes	Yes	Can’t tell	Can’t tell	Can’t tell	Yes	Yes	Can’t tell	Yes	Yes	Can’t tell	No	7/5/1
Gibb et al., 2016 [52]	Yes	Yes	Yes	Can’t tell	Can’t tell	Can’t tell	Yes	Yes	Can’t tell	Yes	Yes	Can’t tell	No	7/5/1
Van Zwanenberg, 2009 [49]	Yes	Yes	Yes	Can’t tell	Can’t tell	Can’t tell	Yes	Can’t tell	Can’t tell	Can’t tell	Yes	Yes	No	6/6/1
Miller et al., 2007 [48]	Yes	Yes	Yes	Can’t tell	Can’t tell	Yes	Yes	Can’t tell	Can’t tell	Can’t tell	Yes	No	No	6/5/2
Buckley et al. 2009 [37]	Yes	Can’t tell	Can’t tell	Can’t tell	Can’t tell	Can’t tell	Can’t tell	Can’t tell	Can’t tell	Can’t tell	Yes	No	No	2/9/2

^a^60% or above

Among the qualitative papers included in the review, two studies met all ten quality criteria on the CASP qualitative checklist while the remaining studies met between nine and three criteria (Table 4). While all ten studies stated a clear objective, included qualitative data that was relevant to the objectives and had an appropriate research design, only two studies considered the relationship between researcher(s) and participants.

Of the five quantitative papers included in the review, there was no study which met all 11 quality criteria based on the CASP cohort study checklist. The studies met between nine and five criteria (Table 5). All studies addressed a clearly focused issue. The lack of consideration of confounding factors was an issue in the studies, as none reflected on this issue.

Among the eight mixed-methods papers included in the review, no study met all 13 quality criteria on the MMAT. Seven studies met between ten and six criteria with one study meeting only two criteria (Table 6). All eight papers stated a clear objective; however, no study considered the potential limitations associated with the integration of qualitative and quantitative data.

### Theoretical underpinnings

As evident from Table 3, the studies included in the review were based on a range of approaches to collectivistic leadership, including shared leadership [39–41, 53, 55], collective leadership [45], distributed leadership [52], collaborative or team leadership [43, 44, 49, 56], co-leadership [46, 54], or a combination of approaches (or used terms were used interchangeably) [38, 42, 47, 48, 50, 51, 57]. Only one included study [37] did not discuss or cite any conceptual framework or theoretical basis for the intervention reported.

### Intervention evaluation

Seven of the included studies were evaluated using a qualitative approach [38, 42, 44, 46, 54–56], six using a quantitative approach [39–41, 43, 45, 57], and eight conducted a mixed methods evaluation [37, 47–53]. Table 3 details the specific evaluation approach for each study. All included studies reported some positive effects of the interventions described, but many studies offered caveats to this; this is further explicated in the narrative review.

### Narrative review

Given the heterogeneity of included papers, studies were therefore categorised by intervention type: co-design interventions, co-leadership interventions, service improvement interventions, team training interventions and individual team development interventions. and are described using a narrative synthesis approach. Co-design interventions are those that involves the equal partnership of individuals to improve efficiency or design interventions or pathways of care. It is a method that is inclusive of various perspectives and employing each other’s knowledge, resources and contributions, to achieve better outcomes or re-design processes for improvement [58]. Service improvement interventions described herein are those that had a clear and explicit focus on enhancing quality and safety outcomes or to fix identified problems in service delivery. Team training interventions are those delivered to and aimed at an entire team to train them together to acquire the same skills and often involved workshops, facilitated learning through seminars and/or learning sets. Finally, the individual team development includes the one study that sought to establish collective leadership in developing and shaping a new team.

### Co-design interventions

Two studies employed co-design approaches which enabled teams to develop solutions to address local problems [39, 40]. Casady and Dowd [40] employed shared leadership approaches to enhance the engagement of staff in participative decision making about initiatives that impact on their jobs in a medical imaging department in the US. Management established new strategic thinking teams in the department which were co-facilitated and led by staff. Each team operated for a 12-week period to address various specific issues relevant to staff and to co-develop solutions. In a similar study, Sanders et al. [39] reported efforts to create a shared leadership model among nurses from various hospital departments where employees were tasked with co-designing and introducing initiatives to make positive changes to the work environment. The former study attributed a reduction in the department’s turnover rate, from 40 to 14.5% within 18 months to this delegation of decision-making and empowerment for improvement across these teams [40]. The turnover rate further declined to 4% after 3 years. The department also observed improvements in employee engagement over this period. In the latter study, it was reported that the new way of working created a more positive work environment and also resulted in reduced staff turnover [39].

### Co-leadership interventions

Four studies reported on the introduction of a co-leadership model, where leadership was shared across two individuals on the team, either a physician-nurse partnership [46, 57], partnership between two nurses [53], or partnership between a nursing manager and a managers with a background in social and welfare-related education [54]. Two studies [53, 57] explored the impact of co-leadership using survey approaches and two employed qualitative interviews to examine the impact of the model [46, 54]. The studies using surveys to evaluate the model achieved high response rates (> 79%) and the qualitative studies each interviewed individuals in eight co-leadership positions. Both studies reported an overall favourable view of the shared leadership approach, particularly among nurses, though one study reported greater scepticism of the model among physicians and therapists [57]. The studies reported that shared leadership increased staff confidence [53, 54] and the model was viewed positively in relation to the dimensions of organisational culture, social interaction, work satisfaction, and shared leadership [53]. It was also found that co-leadership fostered a better working environment for staff [53]. Overall, the results indicated strong support from staff for the model, and in one study 94% of participants responded positively to keeping shared leadership and did not want to return to a traditional single leader model [53].

Role clarity was described as important for the model to be effective [46], the leaders’ personality characteristics [54], knowledge and skills [46], sharing similar values [46, 54], and demonstrating mutual respect [46, 54] were considered important. Good communication [46, 54] and transparency fostered trusting relationships [54]. Successful co-leadership was described as requiring flexibility from leaders engaging alternatively in moments of ‘give and take’ and occasionally stepping back from decision-making and allowing the team to find solutions [54]. From a practical perspective, regular meetings, co-location and organisational support were highlighted as valuable enablers of the co-leadership model [46, 54].

### Service improvement interventions

Three studies describe interventions aimed at sharing responsibility for quality and patient safety outcomes [37, 45, 47]. Two studies adopted mixed methods approaches to evaluation and one used a quantitative assessment. One study explored the introduction of distributed leadership and team working in a therapy department through service re-design [47], another aimed at enhancing quality through building physician-nurse relationships through working with external consultants [37] and the third describes an organisation-level approach to initiative to enhance collective leadership for quality and safety [45]. As a result of these interventions, there was evidence of a flattening of the hierarchy and enhanced collaboration [37, 47], communication [47], mutual support [47], staff satisfaction [37, 45], retention [45], and adoption of leadership responsibilities [47]. Staff perceived the interventions as enabling a more supportive work environment and served to enhance clarity and focus amongst participants [47]. The introduction of the new model was reported to be associated with multiple service quality improvements [45], including a reduction in patient waiting times for therapy [47] and increased patient satisfaction [45, 47].

### Team training interventions

Eleven studies in the review described team training interventions aimed at enhancing collectivistic leadership [38, 41–44, 48–52, 55]. Study designs included quantitative, qualitative and mixed methods studies and a variety of methodologies were employed. Three studies employed a work-based action research approaches [41, 42, 50], two studies utilised case study approaches [38, 44] and two worked with external consultants or coaches [38, 48]. Most interventions included a series of workshops, facilitated sessions or learning sets [41, 42, 48–50] exploring topics including leadership theory, personality profiling, goal setting, communication, conflict management, cognitive instruction, reflection, time and meeting management, performance management, group dynamics, building collaborative relationships, appreciative inquiry, and change management. Other intervention components included developing a team charter [44], introduction of team huddles, mechanisms to encourage feedback, after action reviews to develop shared mental models/vision and standardised communication protocols [52].

Most studies reported moderate to good success in fostering shared leadership behaviours and/or fostering willingness to lead among individuals [41–44, 50–52]. However one study reported that although participants felt the intervention had enabled them to develop as leaders, “the teams all remained convinced that every team needs a leader and felt that leadership was not shared in that sense” ([48]: 34). Similarly, of the four teams involved in another team training intervention, only one successfully transitioned to distributed leadership and effectively collaborating [52]. This suggests such interventions may not work in the same way for all types of teams, yet there was little reflection on why this may be the case.

Other common outcomes of team training interventions were more effective team working [48, 50], evidence of a flattening of hierarchy [49], increased staff engagement [41, 42, 51], greater confidence and empowerment among participants [42, 51], enhanced communication [42–44, 50, 55] and more collaborative problem solving and decision-making [38, 42, 49]. Valuing others’ input and contribution was described as a positive outcome of three studies [38, 50, 55] and greater delegation [42], increased trust [50], and innovation were also reported [44]. Among the recommendations arising from these studies were the need for adequate resources, senior leadership buy-in, physician engagement and continuing education to support shared leadership [55].

### Individual team development interventions

One study evaluated the development of a multidisciplinary team in a Canadian cancer care centre [56]. Consultants were hired to facilitate a series of three, two-day workshops over a three-month period to enhance team leadership development. Interviews were conducted with participants 3 months post-intervention (*n* = 7). Analysis found that, because of the workshops, participants felt they had learned how to communicate, relate to and support one another. There was also an increased sense of solidarity and cohesion among the group. However, the researchers conclude that there is need for on-going team support to maintain gains.

### Overview of drivers of intervention success across studies

Many studies commented on the factors which facilitated the success of their intervention. Physician and senior management support and engagement was purported to play a vital role in success [47, 53, 55]. Studies which included the appointment of two or more leaders to share responsibilities noted that the personal characteristics of the co-leaders impacted on the success of the intervention [37, 45, 46, 53, 54]. Continuous education and communication of outcomes were seen as important in order to internalise shared leadership concepts among staff [55]. This internalisation and engagement from staff was also achieved by interventions which used co-design or co-development which gave team members responsibility in developing the direction and content of the intervention [42, 44, 47]. Finally, providing teams with time and space to discuss the new approach to leadership, how their team works and clarify their goals was considered an important component of successful interventions [47, 48].

## Discussion

This systematic review examined interventions that have been employed with the aim of developing collectivistic approaches to leadership in healthcare settings. Most of the studies included in this review highlight at least moderate success in the enactment of collectivistic leadership approaches and although the studies were heterogeneous, limited initial progress has been made indicating the value of such interventions in healthcare settings. One of the main findings of this review is the paucity of research on interventions to develop collectivistic forms of leadership in healthcare and a concomitant lack of rigor and replication in the field. Additionally, this review highlights the range of approaches to intervention design and varied evaluative methods employed in the field. Consequently, there are no studies included that evaluated the same intervention in the same way, meaning there is a lack of comparability both between and within these categories of studies. This limits the ability to synthesise findings and draw meaningful conclusions from the studies included.

The advancement of field of collectivistic leadership development is inhibited by the variation in study designs and approaches. This is made more problematic by the lack of consensus on the best means of measurement evaluation of collectivistic leadership interventions [1, 59]. Further, the studies in this review were of mixed quality, raising concerns about methodological rigor and completeness of reporting. Due to the small number of papers included however, this review did not exclude papers based on quality appraisal. Journal space constraints and different reporting conventions in various disciplines may explain some of this heterogeneity, as the higher quality of studies included were often dissertations.

There was also heterogeneity observed in the theoretical underpinnings of the interventions/approaches described in the studies, with many studies basing their research on shared leadership, collaborative/team-based leadership models, using multiple approaches or using terms interchangeably. Despite this, it was evident that most of the studies included demonstrated moderate to good success in delivering on the aims of developing collectivistic leadership in healthcare settings. Nearly all studies reported positive outcomes in terms of leadership, engagement, team or organisation performance and specific team-relevant outcomes.

### Intervention content

Team development activities and team training may be required to enable and enhance collectivistic leadership [1], particularly as shared mental models, working towards common goals and role clarity are components of both effective team working and collectivistic leadership approaches. Several studies included components which would be typical of team building and team development programmes [60]. These included interventions related to the development of shared understanding, goal setting, role clarity, communication and recognising competencies among team members [37, 41, 44, 47–49, 56] and interventions which aimed to explicitly value the contribution of others on the team [50, 55]. Many interventions included an aspect of co-design or co-development, whereby team members were given the responsibility to help re-design their service, co-develop their own goals and team charter, or inform the content/direction of the intervention [42, 44, 47]. This was reported to enhance engagement and give ownership to staff, effectively enabling them to take control of, and responsibility for, the intervention.

One crucial feature of many of these interventions was the provision of time and space for teams to physically come together and have dedicated time to reflect on how they do their work and what they are working towards, thus enabling their improved functioning as teams [47, 48]. Increasingly, healthcare is delivered by multidisciplinary teams, with high interdependency between team members and need for co-operation and effective team working to ensure patient safety [61]. Yet, rarely do healthcare teams receive training as a team. As noted by Miller et al., this protected time for team development “was a new experience” for the teams ([48]: 36). Whilst this time to come together as a team may seem a rudimentary or obvious requirement, the reality within which many healthcare teams operate, and the increasing pressures on healthcare services, means that this time is no longer available, and it is considered a low priority for teams to develop elementary team working competencies.

However, these basic team working competencies may be critical to the development of collectivistic leadership in teams. Research by Carson et al. [9] demonstrated that fostering a positive ‘internal team environment’ (that is shared purpose, social support, voice and team trust) had a direct relationship with the emergence of shared leadership in teams and therefore was suggested as an antecedent condition for shared leadership. This is echoed by Yammarino et al. who contend that having these effective team-working competencies in place “should help foster climates where collectivistic leadership may be enacted successfully” ([1]: 399). Given that collectivistic leadership is a relational process where influence and leadership are shared, building interpersonal relationships and shared mental models across the team appears to be an important step to developing collective ways of working in practice.

### Intervention outcomes

As noted previously, all studies included in this review reported positive outcomes and there was some commonality in the type of outcomes reported. In several studies, the decentralisation of power to enable collaborative decision-making and problem solving was associated with increased staff engagement, satisfaction and empowerment [41, 42, 44, 47, 50, 51] and reduced turnover [40]. The shared nature of problem solving and collaborative decision-making were viewed positively by participants in many studies [38, 42, 54, 56], and although described as difficult, the benefits were perceived to outweigh the challenges [54].

Improved communication between team members was a key outcome in several studies, with enhanced communication described both as an enabler of shared leadership [54, 55] and a by-product of the intervention or model [46, 47, 50, 56]. As a result of the interventions implemented, many observed improvements in role and goal clarity and a shared understanding of the work of the team [46, 47, 54]. The effect of working together through the interventions helped to foster mutual respect, trust and support among team members, building cohesion and solidarity [47, 50, 56]. Efforts to explicitly value and recognise the contribution of others resulted in team members feeling valued and feeling their contributions would be welcome and acknowledged [50, 55]. It was reported that individuals felt more self-confident in contributing to the work of the team, in adopting leadership roles and in voicing opinions [48, 51, 53].

There is some evidence from this review that different configurations of collectivistic leadership may be more or less appropriate for various teams. Whilst it was clear many of the interventions demonstrated success in fostering distributed approaches to leadership, these interventions did not always work for every team in the same way [38, 48, 52]. Yammarino et al. [1] suggest that using ‘collectivistic’ leadership as a broad lens through which interventions are evaluated may be more appropriate than one (relatively) narrowly defined theoretical approach.

### Methodological rigour

In several studies included in this review, there was poor reporting of intervention content, little detail on analytical procedures, lack of consideration of potential confounding variables, and gaps evident in the reporting of results. Some studies only collected data post-intervention, resulting in a lack of comparison data and limiting the conclusions that may be drawn. One key concern from the studies in this review is that frequently there was no attempt made to evaluate whether the leadership intervention had been successful in the enactment of collectivistic leadership behaviours. Whilst this was captured or specifically sought in some qualitative and mixed methods studies [48, 50–52], only two quantitative studies measured this [41, 57]. Thus, less than half of included studies assessed the enactment of the desired behaviours post-intervention. Additionally, where there was quantitative measurement undertaken pre- and post-intervention, these studies used bespoke survey instruments to evaluate the intervention, minimising the opportunity for replication and limiting comparability with similar studies [41, 57]. While we acknowledge there is no established valid and reliable instrument that has been well-tested for the purpose of assessing collectivistic approaches to leadership [1, 59], a number of scales have been developed and psychometrically tested and/or have been used in multiple studies [11, 18, 62]. Moving forward, this must be a priority to enable comparability and relative efficacy of interventions aimed at developing collectivistic leadership.

Finally, there was little evidence of the use of a systems lens to fully if or how the intervention may have had a broader impact on the system or other parts of the organisation. Measurement of outcomes was, often, narrowly focused on the team or the specific outcomes of interest and little consideration given to the potential for unintended consequences of interventions.

### Strengths and limitations

A key strength of this review is that it addresses a gap in knowledge and has collated the studies conducted to develop collectivistic approaches to leadership in healthcare settings. The review used both academic and grey literature databases, as well as literature identified by experts, to ensure insofar as possible that publication bias was minimised and that a wide range of studies were considered for inclusion against the eligibility criteria. However, one challenge with this topic is the multitude of terms used to capture collectivistic approaches to leadership and the fragmented and disparate nature of these bodies of literature. However, we hope to have limited the impact of this by looking to previous systematic reviews conducted on collectivistic approaches to leadership from other sectors to help inform the search strategy [14, 16].

Whilst most studies reported positive outcomes in terms of the effect of collectivistic approaches to leadership, an additional limitation of this review is our inability to draw strong causal inferences, based on the relatively small number of studies, heterogeneous nature of the studies, inconsistencies in measurement, and the lack of consideration of confounding variables.

### Recommendations

#### For implementation and practice

There have been calls for the ‘modernisation’ of healthcare, and for more emphasis on training and developmental programmes that facilitate the sharing of leadership and influence among all members of a team [6, 8]. Although tentative, findings from two studies suggest that nurses tend to be more supportive of collectivistic approaches to leadership compared to physicians [57]. This may be due to differences in how these professional groups are trained or how they typically work. Given their influence within the traditional medical hierarchy, several researchers emphasise the necessity of physician engagement in interventions of this nature [47, 53, 55]. Furthermore, studies in this review state that a lack of organisational resources, support, or commitment to collectivistic leadership interventions is a risk to the success of implementation and to sustainability [52, 55]. Thus, it appears for collectivistic approaches to be successful, there is a need to work through and within the relevant hierarchies to first garner support for implementation. Healthcare organisations are typically very hierarchical organisations and operate within traditional ‘command and control’ models of leadership [19]. Therefore, as suggested by O’Toole et al., the notion of shared leadership may initially be “simply counter intuitive” to some ([63]:249) and this lack of familiarity with the concept may help explain some of the scepticism and reluctance to adopt shared models of leadership. Legal issues around accountability may also present challenges, especially to clinical leaders. However, from the studies included, interventions aimed at developing the practice can be effective and can enhance team and organisation performance, once the intervention has organisational support and buy-in from senior management and clinical leaders.

#### Recommendations for research

Several of the authors in this review call for better practices to ensure greater rigor in research of this nature. Recommendations include multidimensional longitudinal designs to ensure measurement at multiple time points to understand impact over time [56], more objective measures of staff engagement, trust and accountability [52], and for research to seek to compare the efficacy of various collectivistic leadership development programmes [51]. Future research must also ensure there is assessment of interventions in terms of the impact on the enactment of collectivistic leadership behaviours and on the healthcare quality and safety. This basic check of intervention validity must be conducted to evaluate if the intervention has been successful in its aims. To realise this, there is a need to address the gap around measurement and working towards common use of a well-tested and psychometrically valid instrument, potentially based on promising initial efforts [17, 18, 64, 65]. Furthermore, the potential unintended consequences of interventions must be considered during evaluation to understand if there may be an impact on other parts of the system.

Surprisingly, none of the studies included in this review employed social network analysis methods in evaluating the impact of interventions. This approach is one that has been advocated and used widely in studies of shared leadership in other settings [14, 59, 66, 67]. Employing the approach in healthcare would offer a useful means of understanding how patterns of leadership and influence may evolve as a result of interventions with teams.

Whilst there is considerable value in establishing commonalities in interventions of this nature, there is also need to consider the balance between the need to compare different collectivistic leadership interventions across different samples and the need to tailor interventions to teams or organisations based on local demands [48, 50]. This will be an important exercise for researchers during implementation in terms of how interventions may be adapted and which elements should be deemed ‘core’ intervention components [68].

It is important to note that empirical research in collectivistic leadership has emerged from various disciplines and this likely accounts for the heterogenous nature of studies in this review. Given that the empirical work in this field is in its infancy, contributions from diverse disciplines enables a breadth and richness of perspectives and approaches as the theoretical work in the field is still developing. Most of the interventions captured in this review can best be described as complex interventions aimed at changing processes and practices to enhance and support collectivistic leadership with these interventions being implemented in complex adaptive health systems [69]. The range of intervention types and methods, on the one hand pose challenges for synthesis and generation of definitive conclusive statements, but on the other hand provide rich insights into how healthcare professionals can and do adopt collectivistic approaches and the many benefits that ensure from this way of working. This review has found little evidence of empirical testing of collective leadership but given that this is an emerging area of scientific inquiry, this is not surprising. In order to advance our understanding of collective leadership it may be necessary to take a broader lens to understanding how teams work as a collective to make things happen, i.e., developing an understanding of the team and collective within complex interventions aimed at the team level. The studies that are included in this review make an important contribution to this understanding. However, as the field of study of collectivistic approaches to leadership matures, the advancement of knowledge and the ability to draw strong conclusions from empirical research will require that researchers reach some consensus on definitions and methods to allow comparability and replicability of studies. We therefore urge researchers in this field to engage in discussion and debate towards informing standardisation of measurement and assessment approaches, adoption of a common framework for complex intervention design and evaluation [70, 71], and the development of some consensus as to the optimal approach or important core intervention components to advance knowledge on how to successfully implement collectivistic approaches to leadership in healthcare settings.

While there is a considerable body of evidence for traditional approaches to leadership, this is not yet the case for non-hierarchical approaches. The shift from one focal leader to collectivistic approaches to leadership will require both a shift in the way individuals and teams are trained and in how performance is evaluated. Beirne [8] suggests that established approaches to leadership development could be reformulated to encourage collective processes that enable a more inclusive and collaborative style and facilitate both formal and informal sharing of leadership. This approach may offer greater opportunities to contrast and compare results with traditional leadership development programmes and their outcomes for teams, but it also runs the risk of continuation of the traditional model of leadership development with the focus remaining on the individual leader. Raelin [72; 4] contends that that if we are to enhance collective leadership in practice, “the entire face of leadership development needs to change” and a different learning model would be required, one characterised by collective and on-going reflection and dialogue. It is also recommended that such interventions should take place in individuals’ work environments [72] to maximise relevance and transferability of learning.

## Conclusions

This systematic review has detailed the intervention content, theoretical underpinning and outcomes of interventions to develop collectivistic approaches to leadership in healthcare settings. It has highlighted the heterogeneity of studies in terms of design, evaluation approach, quality and rigor. Nonetheless, most interventions aimed at introducing collective approaches to leadership demonstrated success with benefits observed in terms of staff engagement, staff satisfaction, and team performance. Whilst this offers a valuable starting point in understanding progress towards the development of interventions to develop collectivistic leadership, there is a need for further research and greater consistency in terms of intervention content and measurement to effectively compare various approaches and draw meaningful conclusions.

## Additional file

Additional file 1: Search engine search strings. Contains the full searches used in each search engine. (DOCX 22 kb)

## Abbreviations

CASP: Critical Appraisal Skills Programme
PROSPERO: Database for protocol details for systematic reviews relevant to health and social care, welfare, public health, education, crime, justice, and international development, where there is a health-related outcome
UK: United Kingdom
US: United States
