# Supplementary material for: Interventions to develop collectivistic leadership in healthcare settings: a systematic review
Source: BMC Health Serv Res. 2019 Jan 25;19:72. doi: 10.1186/s12913-019-3883-x (PMC6347820; doi:10.1186/s12913-019-3883-x)
Supplement: Supplementary file 1 — Search engine search strings. Contains the full searches used in each search engine. (DOCX 22 kb) [file 12913_2019_3883_MOESM1_ESM.docx]

**Additional file 1**

**All searches 01/01/2000 – 08/02/2017**

**PubMed search string**

((((((Team*[Title/Abstract]) OR Group*[Title/Abstract]) AND ( "2000/01/01"[PDat] : "3000/12/31"[PDat] ))) AND ((((((((((((((((Collective leadership[Title/Abstract]) OR Collectivistic leadership[Title/Abstract]) OR Distributed leadership[Title/Abstract]) OR Shared leadership[Title/Abstract]) OR Collaborative leadership[Title/Abstract]) OR Participatory leadership[Title/Abstract]) OR Inclusive leadership[Title/Abstract]) OR Democratic leadership[Title/Abstract]) OR Plural leadership[Title/Abstract]) OR Dispersed leadership[Title/Abstract]) OR Empowering leadership[Title/Abstract]) OR Compassionate leadership[Title/Abstract]) OR Informal leadership[Title/Abstract]) OR Peer leadership[Title/Abstract]) OR Team leadership[Title/Abstract]) AND ( "2000/01/01"[PDat] : "3000/12/31"[PDat] ))) AND ((((((((((((((((((Skill*[Title/Abstract]) OR Intervention*[Title/Abstract]) OR Development[Title/Abstract]) OR Education*[Title/Abstract]) OR Training[Title/Abstract]) OR Strateg*[Title/Abstract]) OR Program*[Title/Abstract]) OR Module*[Title/Abstract]) OR Course*[Title/Abstract]) OR workshop*[Title/Abstract]) OR learning[Title/Abstract]) OR framework*[Title/Abstract]) OR competenc*[Title/Abstract]) OR capabilit*[Title/Abstract]) OR model*[Title/Abstract]) OR curricul*[Title/Abstract]) OR e-learning[Title/Abstract]) AND ( "2000/01/01"[PDat] : "3000/12/31"[PDat] ))) AND (((((((((Health[Title/Abstract]) OR healthcare[Title/Abstract]) OR medical[Title/Abstract]) OR clinical[Title/Abstract]) OR nursing[Title/Abstract]) OR hospital[Title/Abstract]) OR primary care[Title/Abstract]) OR community[Title/Abstract]) AND ( "2000/01/01"[PDat] : "3000/12/31"[PDat] )) Filters: Publication date from 2000/01/01

**PsychInfo and ABI Inform search string (peer-reviewed books, conference papers, journal papers and reports since 2000)**

AB,TI(team* OR group*)

**AND**

AB,TI(Collective leadership OR Collectivistic leadership OR Distributed leadership OR Shared leadership OR Collaborative leadership OR Participatory leadership OR Inclusive leadership OR Democratic leadership OR Plural leadership OR Dispersed leadership OR Empowering leadership OR Compassionate leadership OR Informal leadership OR Peer leadership OR Team leadership)

**AND**

AB,TI(Skill* OR Intervention* OR Development OR Education* OR Training OR Strateg* OR Program* OR Module* OR Course* OR learning OR framework* OR competenc* OR capabilit* OR model* OR curricul* OR e-learning OR workshop*)

**AND**

AB,TI(Health OR healthcare OR medical OR clinical OR nursing OR hospital OR primary care OR community)

**CINAHL search strategy**

TI group* OR AB group* OR TI team* OR AB team*

**AND**

TI Collective leadership OR AB collective leadership OR TI collectivistic leadership OR AB collectivistic leadership OR TI distributed leadership OR AB distributed leadership OR TI shared leadership OR AB shared leadership OR TI Collaborative leadership OR AB Collaborative leadership OR TI Participatory leadership OR AB Participatory leadership OR TI informal leadership OR AB Informal leadership OR TI peer leadership OR AB peer leadership OR TI team leadership OR AB team leadership  OR TI Inclusive leadership OR AB Inclusive leadership OR TI democratic leadership OR AB democratic leadership OR TI Plural leadership OR AB Plural leadership OR TI dispersed leadership OR AB dispersed leadership OR TI empowering leadership OR AB empowering leadership OR TI compassionate leadership OR AB compassionate leadership

**AND**

TI Skill* OR AB Skill* OR TI Intervention* OR AB Intervention* OR TI Development OR AB Development OR TI Education* OR AB Education* OR TI Training OR AB Training OR TI Strateg* OR AB Strateg* OR TI Program* OR AB Program* OR TI Module* OR AB Module* OR TI Course* OR AB Course* OR TI learning OR AB learning OR TI framework* OR AB framework* OR TI competenc* OR AB competenc* OR TI capabilit* OR AB capabilit* OR TI model* OR AB model* OR TI curricul* OR AB curricul* OR TI e-learning OR AB e-learning OR TI workshop* OR AB workshop*

**AND**

TI Health OR AB Health OR TI healthcare OR AB healthcare OR TI medical OR AB medical OR TI clinical OR AB clinical OR TI nursing OR AB nursing OR TI hospital OR AB hospital  OR TI primary care of AB primary care OR TI community OR AB community

**Cochrane**

Team* or group*:ti,ab,kw

**AND**

Collective leadership OR Collectivistic leadership OR Distributed leadership OR Shared leadership OR Collaborative leadership OR Participatory leadership OR Inclusive leadership OR Democratic leadership OR Plural leadership OR Dispersed leadership OR Empowering leadership OR Compassionate leadership OR Informal leadership OR Peer leadership OR Team leadership:ti,ab,kw

**AND**

Skill* OR Intervention* OR Development OR Education* OR Training OR Strateg* OR Program* OR Module* OR Course* OR learning OR framework* OR competenc* OR capabilit* OR model* OR curricul* OR e-learning OR workshop*:ti,ab,kw

**AND**

Health OR healthcare OR medical OR clinical OR nursing OR hospital OR primary care OR community:ti,ab,kw
